# Post-traumatic Epidermal Inclusion Cyst of the Index Finger: A Surgical Case Report

**DOI:** 10.7759/cureus.71883

**Published:** 2024-10-19

**Authors:** Madhu Chandana Reddy Pulakanti, Ayman Nadeem, Mukarram Mohammed Abdul, Manasi Narreddy, Aditi Agarwal, Sana Rahman, Ahmed Aquib Ali, Manisha Bokka, Sreeha Baddam

**Affiliations:** 1 General Surgery, Osmania Medical College, Hyderabad, IND; 2 Internal Medicine, Osmania Medical College, Hyderabad, IND; 3 General Surgery, Nizamabad Medical College, Nizamabad, IND

**Keywords:** acquired dermoid cyst, benign tumor, dermal inclusion, epidermal inclusion cyst, finger trauma, hand surgery, histopathological diagnosis, post-traumatic cyst, soft tissue mass, trauma-induced growth

## Abstract

Epidermoid cysts of the distal phalanx have rarely been reported in practice, although they are known to occur following penetrating trauma and pressure erosion. Notably, the distinction between epidermoid and dermoid cysts is unclear, with both terms being used interchangeably. However, epidermoid cysts are more commonly documented in the literature. We describe the case of a carpenter in his 40s presenting with a lesion on the distal phalanx of his left index finger following a history of trauma to the same area two years ago. This was presumed to be an abscess and incision and drainage were planned. A keratinous epidermoid cyst was diagnosed through histopathological study of the thick-walled cystic structure isolated after dissection. The lesion resolved successfully and healing was uneventful. This report contributes valuable insights into the diagnosis and management of epidermoid cysts of the fingers post-trauma, an underexplored topic in the existing literature.

## Introduction

Epidermoid cysts can develop in various regions, but the involvement of the finger is uncommon. The term has been used interchangeably with dermoid cysts, but while both are cystic choristomas containing cholesterol clefts, keratin, and degenerated blood components, they differ histologically. Epidermoid cysts lack skin appendages on their walls, unlike true dermoids, which contain hair follicles, sebaceous glands, and sweat glands [1]. They are known to occur in the hands at sites of penetrating trauma or pressure erosion [2,3], perhaps due to the implantation of epidermal cells into underlying tissue. Previous reports have suggested the possibility of repeated minor trauma, such as chronic nail biting, rarely leading to cyst formation in the distal phalanx [3]. While the specific question of epidermoid cysts in fingers post-trauma has not been addressed in the available literature, insights have been offered into similar scenarios, and possible management methods. Histopathological confirmation is required for diagnosis, and as a result, the lesions are often misdiagnosed [4,5]. Differentiating dermoid cysts from other malignant osseous lesions by histopathology is vital as benign dermoids can be treated by excision or curettage, while neoplasms may necessitate amputation [4]. This article reports a case of an epidermoid cyst on the volar aspect of the left index finger following trauma and reviews existing literature to provide a commentary on diagnosis and management.

## Case presentation

A 42-year-old male patient, working as a carpenter, presented with swelling on his left index finger for two years. He gave a history of injury, a laceration, to his left index finger while working. The wound was treated by suturing and recovery was uneventful. Four months following, he noticed a small lentil-sized swelling which gradually progressed to the present size, 2x1 cm. It was associated with continuous, stabbing, non-radiating type of pain. There was no associated fever or discharge from the swelling. 

On examination, the skin over the swelling was tense, mildly erythematous but not pinchable. It was tender to touch. There were no sinuses, visible pulsations, or discharge.

**Figure 1:**
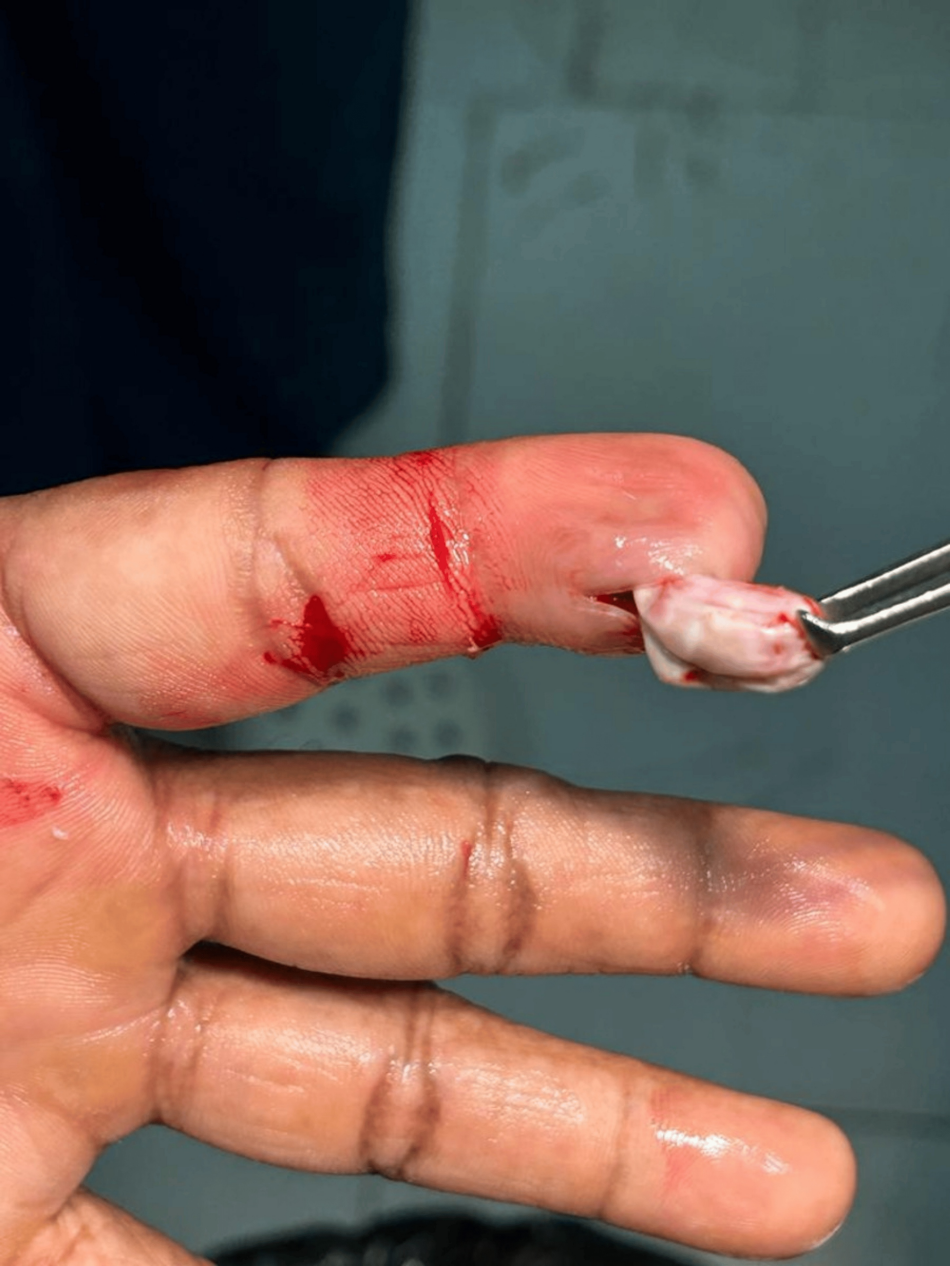
Intraoperative Findings of a Ruptured Cystic Lesion on the Left Index Finger

Based on the findings mentioned, there was a suspicion of an epidermoid cyst following trauma. Consequently, incision and drainage were recommended. A skin incision was made and was deepened. But, instead of pus, a bluish cystic wall was found, which required a revision of our initial diagnosis from an abscess to a cystic swelling. This could be a sebaceous cyst or an epidermoid cyst. The cyst was carefully delineated from its pedicle attachments and adhesions. It was then excised but the wall ruptured during the procedure, disclosing a material with semi-solid consistency as shown in Figure 1. Hemostasis was achieved, and the area was thoroughly washed. The deeper tissues were approximated using 2-0 polyglactin (Vicryl) sutures to prevent the formation of dead space, and the skin was subsequently closed with 3-0 polypropylene (Prolene).

**Figure 2:**
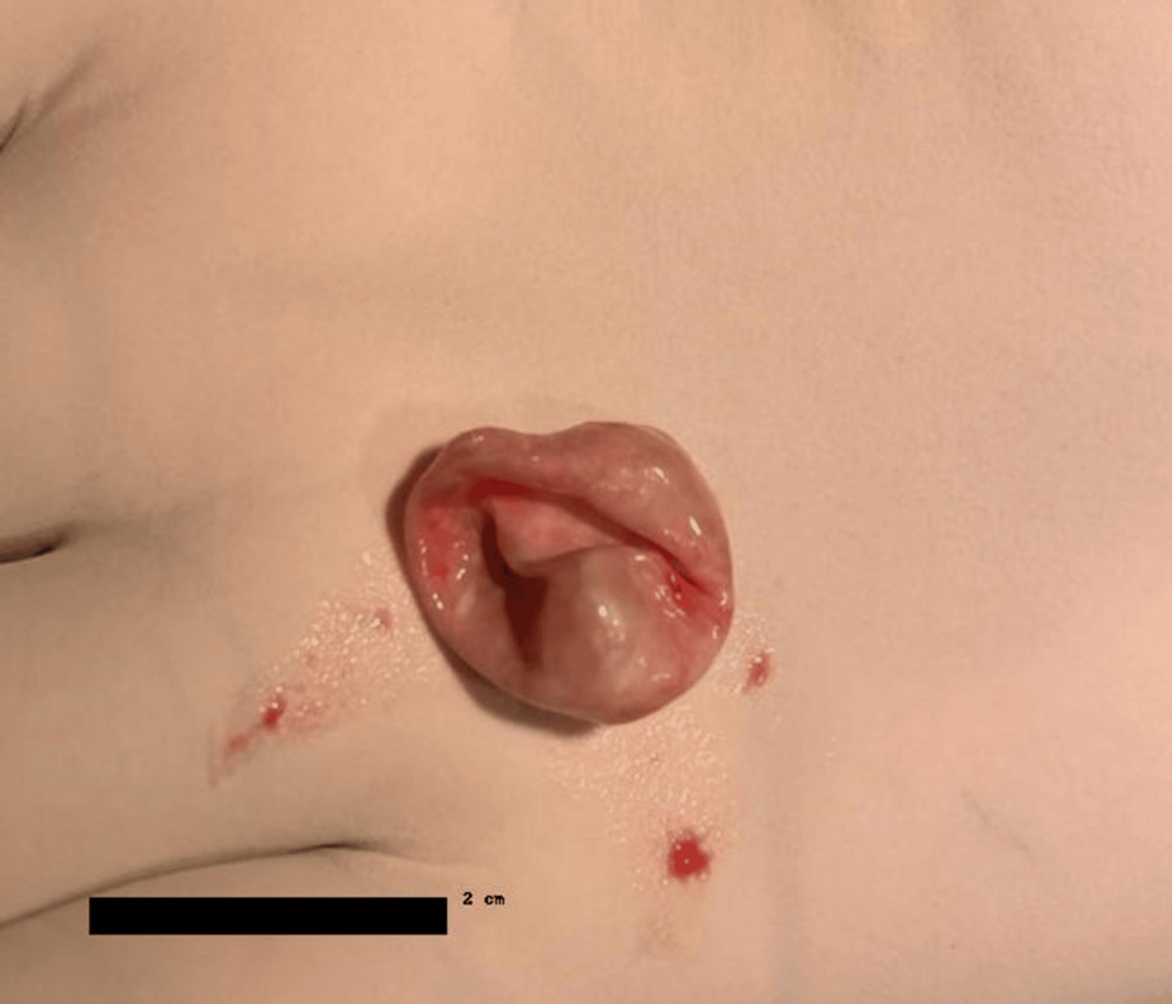
Gross Appearance of the Excised Cystic Mass measuring 2 cm x 1.5 cm.

**Figure 3:**
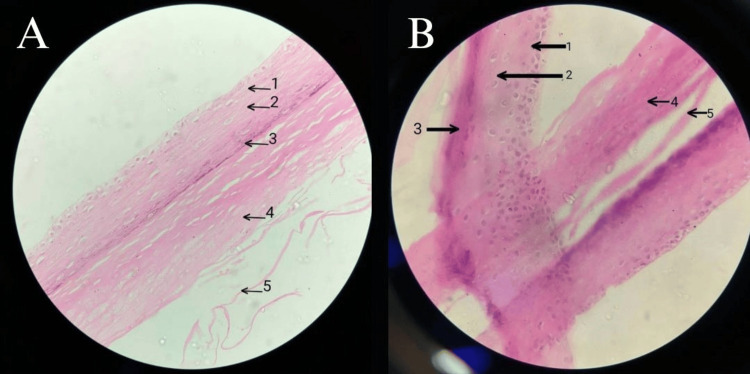
Microscopic Examination of the Cystic Wall Lined with Stratified Squamous Epithelium and Keratin Filled Lumen, Consistent with a Keratinous Epidermoid Cyst (A) Image under Low Magnification (B) Image under High Magnification. 1-Stratum Spinosum  2-Stratum Granulosum  3-Stratum Lucidum  4-Stratum Corneum  5-Lamellated Keratin

The excised mass was sent to histopathology. The gross features consisted of a single cystic gray-brown structure measuring 2 cm x 1.5 cm, and a cut surface showing pultaceous material as shown in Figure 2. Further microscopic studies showed a cystic wall lined with stratified squamous epithelium and a prominent granular layer with a lamellated keratin-filled lumen as shown in Figure 3. These features were consistent with a keratinous epidermoid cyst. Two weeks post-op showed that the wound healed well and the patient was counseled on the possibility of recurrence, though very minimal.

## Discussion

Wernher first reported a case of an epidermoid cyst of the distal phalanx of a finger in 1855 [6]. Worz described the possibility of trauma, including surgery, as a probable cause with a positive history in 24 out of 55 cases reviewed, and more recent reports by Van Tongel et al., Gondal et al., Dumitru et al., and Saraf et al., among others, have corroborated the observed association [3,7-9]. Individuals in occupations that have an increased risk of penetrating injury, such as farmers, factory workers, tailors, and as in our case, carpenters, are therefore more prone to developing acquired epidermoid cysts. Literature suggests these cysts can also occur following exposure to ultraviolet rays, human papillomavirus (HPV), and sometimes following surgical procedures [9,10]. Details from the patient’s history confirm that though the cyst was acquired, it is unclear whether it developed due to the laceration or because of the suturing done to close the laceration. 

Irrespective of the etiology, the deposition of pluripotent surface epithelial tissue in the wound and the accumulation of keratin and epidermal products produced by the epithelial tissue led to the formation of the cyst [9]. Investigations like ultrasonography usually play a relatively smaller role in confirming the diagnosis. More often than not, histopathology is crucial in determining the exact nature of the swelling to give a final diagnosis. In this instance, the presence of a cystic wall lined with a granular, stratified squamous epithelium and a lumen filled with lamellated keratin has afforded us a lucid comprehension of the pathophysiology underlying the observed swelling. Excision remains the only standard treatment. In a literature review of sublingual epidermoid cysts, it was noted that complete surgical excision accounts for low to almost nil recurrence rates [11]. Therefore, it can be inferred that significant research is needed to know the proper etiology and to develop effective preventive strategies to reduce its incidence.

## Conclusions

This report presents a case involving an epidermoid cyst of the index finger following trauma, which represents a typical yet frequently disregarded consequence of skin injury. This emphasizes the significance of identifying such lesions within post-traumatic soft tissue masses. Precise imaging and histopathological analysis are imperative for distinguishing epidermoid cysts from other cystic lesions and neoplasms. Given the potential for misdiagnosis, it is essential for clinicians to be cognizant of this condition and to refrain from unnecessary investigations to ensure the provision of optimal patient care.
